# Investigation of a SARS-CoV-2 Outbreak at an Automotive Manufacturing Site in England

**DOI:** 10.3390/ijerph19116400

**Published:** 2022-05-24

**Authors:** Amber I. Raja, Karin van Veldhoven, Adanna Ewuzie, Gillian Frost, Vince Sandys, Barry Atkinson, Ian Nicholls, Alice Graham, Hannah Higgins, Matthew Coldwell, Andrew Simpson, Joan Cooke, Allan Bennett, Chris Barber, Derek Morgan, Christina Atchison, Chris Keen, Tony Fletcher, Neil Pearce, Elizabeth B. Brickley, Yiqun Chen

**Affiliations:** 1 Health Equity Action Lab, Department of Infectious Disease Epidemiology, London School of Hygiene and Tropical Medicine, London WC1E 7HT, UK; amber.raja@lshtm.ac.uk (A.I.R.); adanna.ewuzie@lshtm.ac.uk (A.E.); elizabeth.brickley@lshtm.ac.uk (E.B.B.); 2 Department of Non-Communicable Disease Epidemiology, London School of Hygiene and Tropical Medicine, London WC1E 7HT, UK; karin.van-veldhoven@lshtm.ac.uk; 3 Science Division, Health and Safety Executive, Buxton SK17 9JN, UK; gillian.frost@hse.gov.uk (G.F.); vince.sandys@hse.gov.uk (V.S.); matthew.coldwell@hse.gov.uk (M.C.); andrew.simpson@hse.gov.uk (A.S.); joan.cooke@hse.gov.uk (J.C.); chris.barber@hse.gov.uk (C.B.); derek.morgan@hse.gov.uk (D.M.); chris.keen@hse.gov.uk (C.K.); 4 Research and Evaluation, UK Health Security Agency, Porton Down, Salisbury SP4 0JG, UK; barry.atkinson@phe.gov.uk (B.A.); ian.nicholls@phe.gov.uk (I.N.); allan.bennett@phe.gov.uk (A.B.); 5 Rapid Investigation Team, Field Services, UK Health Security Agency, Wellington House, London SE1 8UG, UK; alice.graham@phe.gov.uk (A.G.); hannah.higgins@phe.gov.uk (H.H.); christina.atchison@phe.gov.uk (C.A.); 6 Chemical and Environmental Effects Department, UK Health Security Agency, Chilton OX11 0RQ, UK; tony.fletcher@phe.gov.uk; 7 Department of Medical Statistics, London School of Hygiene & Tropical Medicine, London WC1E 7HT, UK; neil.pearce@lshtm.ac.uk

**Keywords:** SARS-CoV-2, COVID-19, workplace, outbreak, manufacturing

## Abstract

Workplace-related outbreaks of severe acute respiratory syndrome coronavirus 2 (SARS-CoV-2) continue to occur globally. The manufacturing sector presents a particular concern for outbreaks, and a better understanding of transmission risks are needed. Between 9 March and 24 April 2021, the COVID-19 (coronavirus disease 2019) Outbreak Investigation to Understand Transmission (COVID-OUT) study undertook a comprehensive investigation of a SARS-CoV-2 outbreak at an automotive manufacturing site in England. The site had a total of 266 workers, and 51 SARS-CoV-2 infections. Overall, ventilation, humidity, and temperature at the site were assessed to be appropriate for the number of workers and the work being conducted. The company had implemented a number of infection control procedures, including provision of face coverings, spacing in the work, and welfare areas to allow for social distancing. However, observations of worker practices identified lapses in social distancing, although all were wearing face coverings. A total of 38 workers, including four confirmed cases, participated in the COVID-OUT study. The majority of participants received COVID-19 prevention training, though 42.9% also reported that their work required close physical contact with co-workers. Additionally, 73.7% and 34.2% had concerns regarding reductions in future income and future unemployment, respectively, due to self-isolation. This investigation adds to the growing body of evidence of SARS-CoV-2 outbreaks from the manufacturing sector. Despite a layered COVID-19 control strategy at this site, cases clustered in areas of high occupancy and close worker proximity.

## 1. Introduction

Coronavirus disease 2019 (COVID-19), caused by severe acute respiratory syndrome coronavirus 2 (SARS-CoV-2), has had a devastating impact on the lives and livelihoods of people globally. Although the proportion of individuals working-from-home has increased during the pandemic [1], essential workers have been required to be on-site, putting them at an increased risk of exposure to SARS-CoV-2 and severe COVID-19 compared to non-essential workers [2,3]. Workplace outbreaks of SARS-CoV-2 continue to occur, with specific sectors including manufacturing being particularly impacted [4,5,6].

**Figure 1:**
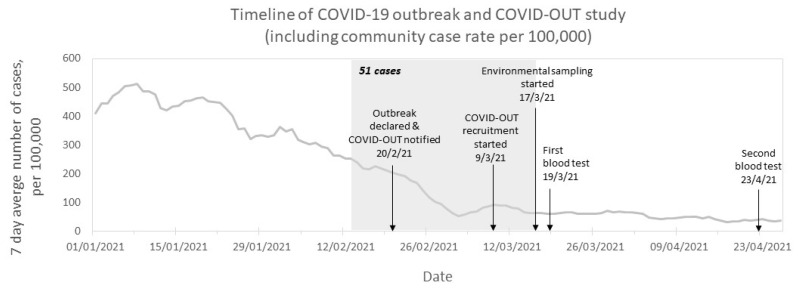
Timeline and epidemiological curve of COVID-19 outbreak investigation in a UK automotive manufacturing site between 13 February 2021 and 23 April 2021. Arrows indicate key dates of the outbreak and COVID-OUT study. The grey box indicates the period of time during which 51 acute SARS-CoV-2 infections were reported by the company. The line chart represents the 7-day case rate for the lower tier local authority area (LTLA) of the site (contains public sector information licensed under the Open Government Licence v3.0 from [10]; the date of download: 18 August 2021).

As part of the PROTECT COVID-19 National Core Study [7], the COVID-OUT (COVID-19 Outbreak investigation to Understand Transmission) study aims to provide a better understanding of workplace outbreaks and transmission risks across different work sectors. Here, we report an outbreak investigation of a cluster of SARS-CoV-2 cases at an automotive manufacturing site in England, United Kingdom (UK). The outbreak was declared on 20 February 2021 by Public Health England (PHE; now UK Health Security Agency, UKHSA), during the third national lockdown (6 January to 7 March 2021 [8]). Government instructions required individuals to stay home unless shopping for essentials, exercising, seeking medical assistance, escaping domestic abuse, or going to work if working-from-home was not possible [9]. Case rates of SARS-CoV-2 in the local area had been decreasing since January 2021 (Figure 1), in line with national trends [10]. An initial investigation (unpublished internal report) conducted by PHE, identified the Alpha strain of SARS-CoV-2 (lineage: B.1.1.7, variant: VOC-20DEC-01) among cases from the outbreak. Our subsequent investigation assessed worker- and workplace-associated risk factors, which may have contributed to the transmission of SARS-CoV-2.

## 2. Materials and Methods

An initial cluster of 26 SARS-CoV-2 cases at an automotive manufacturing site was notified to the PHE local authority on 13 February 2021. Following the declaration and notification of the outbreak by the local PHE Health Protection Team on 20 February 2021, the COVID-OUT team undertook a follow-up investigation from 9 March to 23 April 2021, using a previously described protocol [11]. Ethical approval was provided by the NHS North East Research Ethics Committee (Reference 20/NE/0282).

Participants recruited into the study were asked to complete a detailed questionnaire at baseline (full questionnaire available at [12]), which comprised a total of 67 questions and collected information on their work, lifestyle and health. Additionally, the completion of three shorter follow-up questionnaires was requested at weeks two, three and six.

Participants also underwent SARS-CoV-2 testing (i.e., by self-administered nose and throat swabs, and blood samples collected by phlebotomy). Viral ribonucleic acid (RNA) testing was conducted on nose and throat swabs at baseline and weeks two and three using the Roche cobas^®^ SARS-CoV-2 assay. Samples yielding crossing threshold (Ct) values of <35 were assessed for whole genome sequencing (WGS). Antibody testing was conducted on blood samples collected at baseline (19 March 2021) and week six (23 April 2021), using the Roche Elecsys^®^ (Basel, Switzerland) Anti-SARS-CoV-2 spike (S) and nucleocapsid (N) binding assays. All assays were performed at the PHE Porton Down Laboratory. Confirmed cases were defined as participants who presented during the outbreak period with: (i) real-time polymerase chain reaction (RT-PCR) evidence of a SARS-CoV-2 infection, (ii) N-specific seroconversion, or (iii) self-reporting of a positive test (i.e., by RT-PCR or lateral flow device [LFD]) with positive N antibody results. Suspected cases were defined as participants who had no positive RT-PCR or N antibody results from the COVID-OUT testing, but who presented during the outbreak period (13 February to 17 March 2021) with: (i) a self-reported positive test (i.e., by RT-PCR or LFD) or (ii) symptoms consistent with COVID-19 defined as: (a) acute onset of fever (>37.8 °C) and new continuous cough or (b) acute onset of any three or more symptoms of fever (>37.8 °C), cough, shortness of breath, loss of taste or smell, runny nose, fatigue, sore throat, muscle or body aches, headache, nausea or vomiting, and/or diarrhoea.

An environmental assessment [13] was conducted on 17 March 2021, which included collecting information on the building layout, ventilation, temperature, humidity, air movement and noise levels, workforce information (e.g., shift patterns), and worker observations (e.g., adherence to infection control measures and worker interactions). Surface sampling for viral RNA [11] was conducted at the same time, with collected samples sent to the PHE Porton Laboratory for nucleic acid extraction using the Viral RNA Mini Kit (Qiagen, Germany) followed by RT-PCR analysis using the CerTest Biotec Viasure (Zaragoza, Spain) two-target N and ORF1ab assay. PCR grade water was used for a negative extraction control sample and as a no-template control for RT-PCR; positive RT-PCR control material was supplied in the Viasure RT-PCR kit. Confirmed positive samples were those with both replicates testing positive for at least one target, and suspected positive samples were those with a single replicate testing positive for at least one target. Samples with Ct values of ≤35 were further analysed for WGS. Spot carbon dioxide (CO_2_; used as a proxy for ventilation), humidity, and temperature measurements were taken on the day of the site visit, as well as longitudinally, in various locations around the site. Longitudinal measurements were taken between 26 March and 22 April for CO_2_, and between 17 March and 8 April for temperature and humidity. Carbon dioxide levels of 1500 ppm or more were considered indicative of inadequate ventilation [14].

Attack rates were calculated by dividing the number of cases by the total number of workers [15] using company-supplied data. Statistical analyses were conducted using Stata/SE (version 17.0) and R (version 3.6.2). An odds ratio was used to compare the relative frequency of cases between working zones. A Chi-squared test was used to compare the attack rate at the site with the cumulative incidence in the local community.

## 3. Results

From 13 February to 17 March 2021, a total of 51 out of 266 workers (attack rate of 19.2%) were reported by the company to have SARS-CoV-2 infections. Two-hundred-and-sixty-four workers (excluding two contract cleaners) from the site were invited to participate in the study between 9 and 19 March. Thirty-eight workers (52.9% male; mean age, range = 39.7, 21 to 64 years; a response rate of 14.4%; Table S1) were included in the COVID-OUT study (Figure S1). Of these, four participants (75.0% male; mean age, range = 52.7, 41 to 64 years) were confirmed cases who self-reported positive SARS-CoV-2 tests (*n* = 3 RT-PCR and *n* = 1 LFD) during the outbreak period and tested positive for both N- and S-specific antibodies against SARS-CoV-2 during the COVID-OUT study. The remaining thirty-four participants (47.4% males; mean age, range = 38.8, 21 to 54 years) were identified as non-cases, including two participants who reported testing positive for SARS-CoV-2 in December 2020 (i.e., prior to the start of the outbreak). No suspected cases were identified (0/38).

Participants reported working in production lines, assembly, and finishing (63.2%), in offices (23.7%), in material and preparation (5.3%), and in all areas (5.3%; Table 1). Thirty-five (94.6%) participants reported working indoors, of whom 34.6% (9/26) reported access to fresh air whilst working, 23.1% reported no access to fresh air, and 42.3% reported that they did not know. A total of 60% of respondents worked with six or more contacts whilst indoors, with 42.9% indicating that their work required close physical contact and 18.2% were rarely socially distanced from colleagues. The majority of participants (77.8%) felt they regularly had to talk loudly or to lean in to listen and speak to people at work, with 70.8%, for whom it was applicable, reporting that there were no dividers between themselves and their colleagues. Despite 97.4% of participants being on permanent work contracts, 62.2% (23/37) thought their pay would decrease due o, 73.7% (28/38) were worried about reduced income in the future, and 34.2% (13/38) had concerns about unemployment in the future if they had to self-isolate due to COVID-19.

**Figure 2:**
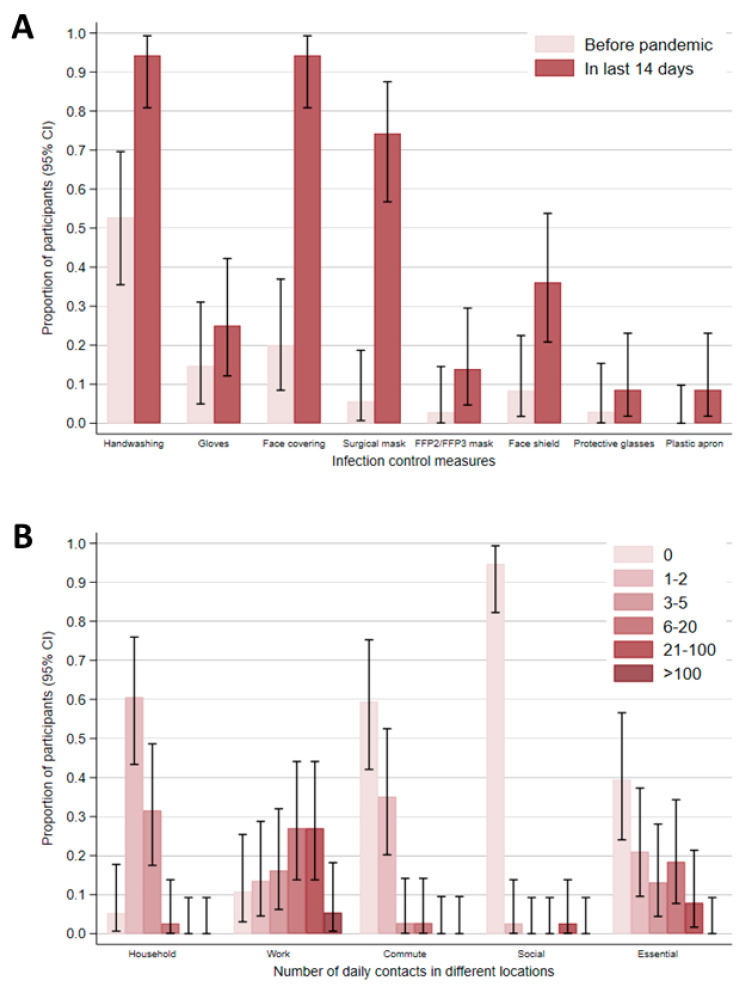
**Baseline questionnaire responses of participants from an automotive manufacturing site—England, United Kingdom**. (**A**) Proportion of participants reporting infection control measures at workplace before the pandemic and within 14 days prior to completing the questionnaire. (**B**) Proportion of participants reporting their daily number of contacts in different locations within the last 14 days of completing the questionnaire. Error bars represent 95% confidence intervals (CI). The following data were missing: handwashing before pandemic (*n* = 2) and in last 14 days (*n* = 3), gloves before pandemic (*n* = 4) and in last 14 days (*n* = 2), face covering before pandemic (*n* = 3) and in last 14 days (*n* = 3), surgical mask before pandemic (*n* = 2) and in last 14 days (*n* = 3), FFP2/ FFP3 mask before pandemic (*n* = 2) and in last 14 days (*n* = 2), Face shield in pandemic (*n* = 2) and in last 14 days (*n* = 2), protective glasses before pandemic (*n* = 4) and in last 14 days (*n* = 3), plastic apron before pandemic (*n* = 2) and in last 14 days (*n* = 3), household contacts (*n* = 0), work contacts (*n* = 1), commute contacts (*n* = 1), social contacts (*n* = 0), and essential contacts (*n* = 0).

Participants were asked about infection control measures and their contact patterns at and outside of work. With the exception of one participant, all other participants with available data reported having received training (i.e., reading guidance and/or formal training) about preventing COVID-19 transmission in their workplace (97.3%; Table 1). Overall, participants reported high uptake of infection control measures within the last 14 days of completing the baseline questionnaire, including increased handwashing and use of face coverings compared to the pre-pandemic period (Figure 2A). Of note, 74.3% (26/35) and 13.9% (5/36) of participants reported use of surgical and FFP2/ FFP3 masks in the last 14 days, respectively. Participants also indicated that their workplace had hand washing/ sanitising facilities (100%) and signage for good hand hygiene practice (100%). Despite this, 50% of confirmed cases, but none of the non-cases, self-reported never washing or sanitising their hands at work. Overall, participants reported higher numbers of contacts at work and during essential activities (e.g., food shopping or visiting GP), with a limited number of contacts at home, whilst commuting, and during social activities (e.g., going to restaurants) (Figure 2B). No domestic or international travel was reported.

All four confirmed cases reported the presence of symptoms compatible with COVID-19, and three reported three or more symptoms. Symptoms included loss of taste (4/4; 100%), dry cough (3/4; 75%), fever (2/4; 50%), productive cough (1/4; 25%), and shortness of breath (1/4; 25%). Although none of the confirmed cases reported working with a symptomatic or positive contact, 75% reported living with a symptomatic or positive contact. Of note, 66.7% of confirmed cases and 21.9% of non-cases lived with a co-worker (Table S1). Additionally, 26.5% and 17.7% of non-cases reported working with a positive or a symptomatic contact, respectively, although all cases in the COVID-OUT study reported that they stopped working at the time of symptoms. None of the cases had been vaccinated at the time of the COVID-OUT baseline questionnaire, whereas 11.8% (4/34) non-cases who disclosed their vaccination status had received a COVID-19 vaccine (*n* = 3 Oxford/AstraZeneca and *n* = 1 Pfizer-BioNTech; Table 1). Notably, the overall vaccination rate among participants was only 10.5% (4/38) compared to 43.3% in the local community, which may, in part, reflect differences in the age distributions.

**Figure 3:**
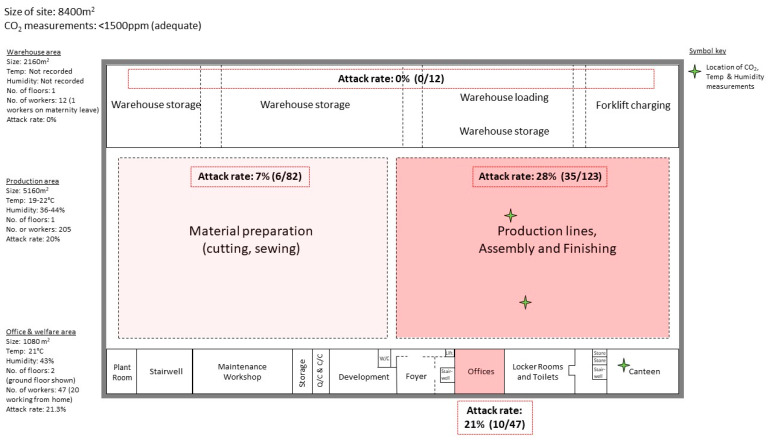
**Site floor plan of the automotive manufacturing site—England, United Kingdom.** Site divided into three main sections: warehouse, production, and office and welfare (e.g., canteen). The office and welfare section is split across two floors and only the first floor is shown in the figure. The second floor contains offices (enclosed and open-plan), meeting rooms, toilets, and small canteen. The attack rate for specific areas was calculated by the number of positive cases in area divided by the total number of workers for the area. The volumes have been approximated for each section and do not account for the slopes of the ceilings. Abbreviations: Quality control (QC), Climate control (CC), W/C (Water closet), and Temperature (Temp).

An environmental assessment was carried out at the manufacturing site, which occupied an 8400 m^2^ building and housed 266 workers (235 permanent employees, 29 agency staff, and 2 contracted members of cleaning staff). The building was divided by walls into three main sections: warehousing (*n* = 12 workers; 5.6 persons/1000 m^2^), an open-plan production section (*n* = 205 workers; 39.7 persons/1000 m^2^) including material preparation (*n* = 82 workers) and production, assembly, and finishing (*n* = 123 workers) areas, and an office and welfare section (*n* = 47 workers; 43.5 persons/1000 m^2^; Figure 3). An overall attack rate of 19.2% (51/266 workers) from 13 February to 17 March 2021 was provided by the company and was significantly higher than the cumulative incidence in the local area during the same period (*p* < 0.0001, Chi-squared; Figure 1). The attack rate at the site differed across the three main work sections. The highest attack rate was among office workers (21.3%,10/47); notably, 20 office workers were reported by the company to engage in work from home on some working days. The attack rate in the production section was 20% (41/205) overall, but varied between 28% in the production lines, assembly, and finishing area and 7% in the material preparation area (odds ratio, 95% CI: 0.20, 0.07 to 0.52). There were no cases from the warehouse, thus this section had an attack rate of 0%.

The method of ventilation varied for different sections of the site. The warehouse had no air conditioning or heating system. Instead, the area was naturally ventilated as doors were nearly always open to allow access for vehicles. This, together with the small number of workers present in this large area, meant that no additional ventilation was recommended. No humidity or temperature measurements were taken in the warehouse, but the COVID-OUT team noted that this section was markedly cooler than the rest of the site. The main production section had no mechanical general ventilation system and the doors to this area were predominantly closed. However, some air movement was provided by a bank of six open-faced spray booths located in the centre of this section, though this was not their primary purpose. Longitudinal CO_2_ measurements taken at Lines 1 and 2 in the production lines, assembly, and finishing area (Figure 3) remained below 1000 ppm, indicating that the ventilation rates in this section were adequate. The mean relative humidity around Line 1 was 40% (range 23–57%) and Line 2 was 35% (range 17–58%). The section was heated by an under-floor system, and the temperature ranged between 16–28 °C in this area. In the office and welfare section, ventilation was provided by an in-ceiling Lossnay heat exchange system, with outside condenser units. The ventilation system did not recycle air. A building management system was used to control and monitor the environment. Longitudinal CO_2_ measurements were taken in the canteen (Figure 3), which remained below 600 ppm, indicating that the ventilation in this area was adequate. The mean relative humidity in the canteen was 41% (range 17–68%) and the temperature ranged between 16–25 °C. Overall, the assessment of CO_2_ at this site did not identify any areas where CO_2_ levels were sufficiently elevated to suggest a problem with ventilation.

The environmental assessment also included surface sampling of work (e.g., benches and computers) and communal (e.g., lockers and vending machines) areas, and high-touch surfaces (e.g., tap handles) for viral RNA. A total of 36 samples were collected from across the site; with 14 (38.9%) samples confirmed positive for SARS-CoV-2 RNA and 3 samples (8.3%) identified as suspected positive (Table 2). The level of RNA detected was typically very low (Ct value > 35.0); although, 2 samples from the male locker room produced Ct values between 32–35. The COVID-OUT team noted that the lockers were poorly maintained, and this may have contributed to the levels of RNA detected. These 2 samples were sent for WGS but did not pass quality control, indicating that the genomic material was highly degraded.

During the environmental assessment, the COVID-OUT team also observed and collected information on workplace and worker practices. In response to the pandemic, and prior to the outbreak at the site, the company had already implemented a number of infection control measures in adherence to government guidance. Office-based workers were encouraged to work from home three days a week to limit the number of workers in the offices, and work meetings were held virtually on company-purchased software rather than face-to-face. A full site shutdown occurred for a month between March and April 2020, and a phased return from furlough was adopted upon reopening of the site, with workers receiving a full induction on new infection control procedures. The company had secured large stocks of personal protective equipment (PPE; e.g., face shields), face masks and hand sanitiser, as well as a guaranteed supply of soap. Handwashing/sanitising and PPE stations were available to all workers, as well as visitors. However, these stations were not placed close to all the regularly used entrance–exit points of various areas/rooms. In the main production section, 2 m spacing was in place and tasks requiring closer working proximity were avoided wherever possible. The company issued mandatory face coverings (e.g., face shields or masks) to all employees, although workers used a range of different face covering (e.g., including cloth masks). All staff and visitors had their temperatures checked upon arrival to the site. Visitors, including inter-company workers, had restricted access, with all visitors requiring an entry pass for the site, as well as a site-specific induction. Inter-company travel also required executive approval.

In response to the outbreak, the company initially introduced daily LFD testing for workers, which was reduced to weekly testing by the time of the COVID-OUT team visit, and an internal track and trace system. Temperature checks were increased to three times per day. To prevent sick workers from attending work, office workers received sick pay, and production and warehouse workers received statuary sick pay after three days of sick leave. There were signs to reinforce social distancing and one-way systems were developed. Already mandatory face masks were checked daily, and workers were advised not to share equipment.

In the production section, observation of working practices showed that although signage and markings to address social distancing were in place, 2 m distancing was not always maintained. Screens had been erected between workers facing each other but there were no screens between those working side-by-side. The average noise levels measured at the production lines were approximately 70 dB (A) and were not considered sufficient to require raised voices or for workers to be less than 2 m apart for effective communication. When both physical barriers and social distancing were not possible, teams were also organised into “bubbles”. However, this did not include office workers, who regularly visited and interacted with workers from the production lines, assembly, and finishing area.

Other areas, such as the canteen, were also rearranged to allow for social distancing, with screens at shared tables, floor markings and removal of seating. For instance, a table of four had two seats removed and the remaining two were placed diagonally. The reduced seating allowed for a maximum of 20 workers to be seated and may have encouraged staggering of workers’ breaktimes and promoted social distancing. In the canteen, there were also signs reminding workers to wipe surfaces with cleaning wipes provided, although the COVID-OUT team noted that these wipes were limited (i.e., not on every table or adjacent to vending machines). Measures to minimise overcrowding in the locker rooms and toilets included blocking off adjacent sinks, urinals, and cubicles, though no maximum occupancy was mandated for these facilities. The locker rooms and toilets were noted to be potential pinch points, as there was only one entrance–exit; and although no overcrowding was seen, this area had the potential to become overcrowded at the beginning and end of shifts.

After the outbreak, the cleaning of all facilities, including high-touch points (e.g., toilets, door handles, canteens) was increased. Additional cleaning of work areas of symptomatic workers was arranged using hotspot maps of cases to inform the cleaners. Following the surface sampling findings of low levels of viral RNA (e.g., male locker room) by COVID-OUT, twice daily routine cleaning was implemented in this area to minimise transmission risk.

## 4. Discussion

A better understanding of SARS-CoV-2 transmission in occupational settings is essential to safeguard workers, their livelihoods, and the wider economy. We investigated an outbreak of SARS-CoV-2 at an automotive manufacturing site in England, which occurred during the third national lockdown and during a period of decreasing infection rates in the local area. The attack rate for this outbreak suggests an increased risk of infection compared to the local community case rates, as has been observed for other workplace outbreaks [16].

The attack rate at this site varied depending on the area of work. Transmissibility of SARS-CoV-2 is dependent on multiple factors, including environmental factors and those associated with the type of work being conducted [17]. Although environmental factors such as humidity, temperature and ventilation were not of concern at this site, other sites in the manufacturing sector may require workers to work in refrigerated or humid conditions, which may impact the use of PPE and face coverings [6] as well as viral transmission [18]. Tasks requiring workers to be in close proximities to each other for prolonged periods also increase the likelihood of viral transmission [19]. At this site, the highest attack rate was in the production, assembly, and finishing area, which had the greatest number of workers and required close proximity working over extended periods. The attack rate of office workers was similar to the main production section, even though office workers were encouraged to work from home three times a week, and may be due to office workers regularly interacting with workers from the main production section and not being part of the ‘bubble’ system. Additional infection control measures could include the use of screens between workers working side-by-side, limiting face-to-face interactions between workers from different work sections, and including office workers in the ‘bubble’ system with production workers. The attack rate of warehouse workers was 0% and was likely due to the limited number of workers in this section and the constant natural ventilation.

The company had implemented many infection control procedures before and after the outbreak. Overall adherence to infection control procedures, including the use of face coverings, at this workplace was high. However, with half of the confirmed cases reporting poor hand hygiene practices after the outbreak, additional handwashing/sanitising stations and refresher COVID-19 prevention training may be required. High uptake of surgical mask use was reported among participants, although the use of FFP2/FFP3 masks remained limited; a recent test-negative design study comparing the odds of testing positive for SARS-CoV-2 by the type of face covering used indoors found the lowest odds of testing positive for SARS-CoV-2 among individuals using FFP2/FFP3 masks [20]. Additionally, the company conducted daily checks on the use of face masks; however, compliance with their proper use throughout the day cannot be confirmed. Furthermore, although the majority of participants were on permanent work contracts, there were still concerns about the possible detrimental financial impact of self-isolation that may have led to presenteeism, as indicated by the approximate one-quarter of participants who reported working with a positive contact. Additionally, the COVID-19 pandemic has heightened financial concerns and has seen an increase in adverse mental health implications [21]. To mitigate these concerns, there needs to be clear communication about the support available to workers, as well as an equitable sick pay policy.

Our participant data suggest that there was good adherence to government lockdown guidance outside of work at the time of the study, with participants reporting limited social activities and interactions. However, social desirability bias could lead to an overestimation of participants reporting adherence to infection control measures. Although the majority of confirmed cases from our study reported living with a positive symptomatic individual, we could not ascertain whether the study cases or their household contacts had the symptoms or tested positive first. Other limitations of our study include a low worker participation rate of 14.4%, with an underrepresentation of warehouse workers. The small sample size also limited the assessment of individual risk factors. To overcome this limitation and encourage the participation of workers in the study, financial incentives have been applied to later outbreak sites, though the impact of this incentive has not yet been assessed. Additionally, due to changes in the cleaning regimen between the outbreak and the timing of our investigation, only limited viral material was recovered from the site, which prevented the identification of work areas highly associated with the outbreak and the confirmation of viral strain.

## 5. Conclusions

Manufacturing may represent a particularly vulnerable work sector for SARS-CoV-2 outbreaks, with it being a major site of workplace outbreaks in other countries [4,5,6]. This sector largely requires on-site work, often in close proximity to co-workers for extended periods of time, with multiple production line workers handling the same materials and where social distancing is not always possible. This is substantiated by the findings of our investigation at an automotive manufacturing site, which highlights that despite the implementation of a layered COVID-19 control strategy at this site, cases cluster in areas of high occupancy and close worker proximity. Additionally, COVID-19 isolation and work closure policies have fueled the financial concerns of workers in this sector. To overcome these challenges and safeguard the lives and livelihoods of workers, additional guidance and support are needed for this work sector. Our approach of combining data collected from participating workers as well as on-site environmental assessment and sampling provides insights into potential risk factors of this SARS-CoV-2 outbreak and areas of potential improvement to minimise workplace transmission risks. Our investigation at an automotive manufacturing site adds to the growing body of evidence of outbreaks in the manufacturing sector globally.

## Figures and Tables

**Table 1:** Participant work factors and COVID-19 prevention related responses to baseline COVID-OUT study questionnaire.

		Non-Cases (*n* = 34)	Cases (*n* = 4)	Total (*n* = 38)
**Work factors**				
Work area	All areas	2 (5.9)	0	2 (5.3)
	Materials and preparation	2 (5.9)	0	2 (5.3)
	Office	4 (11.8)	1 (25)	5 (13.2)
	Office + other areas	4 (11.8)	0	4 (10.5)
	Production, assembly, and finishing	21 (61.7)	3 (75)	24 (63.2)
	Unknown	1 (2.9)	0	1 (2.6)
Employment contract	Permanent	33 (97.1)	4 (100)	37 (97.4)
	Zero hours	1 (2.9)	0	1 (2.6)
Same shifts	No	2 (6.3)	0	2 (5.6)
	Yes	30 (93.8)	4 (100)	34 (94.4)
	Missing	2	0	2
Work indoors	No	1 (3)	1 (25)	2 (5.4)
	Yes	32 (97)	3 (75)	35 (94.6)
	Missing	1	0	1
No. of contacts whilst working indoors	Alone	3 (13.6)	0	3 (12)
	1–2	1 (4.5)	1 (33.3)	2 (8)
	3–5	5 (22.7)	0	5 (20)
	≥6	13 (59.1)	2 (66.7)	15 (60)
	Missing	11	0	11
	Not applicable	1	1	2
Indoor fresh air	No	6 (26.1)	0	6 (23.1)
	Yes, mechanical	5 (21.7)	0	5 (19.2)
	Yes, opening window/door	2 (8.7)	0	2 (7.7)
	Yes, other	2 (8.7)	0	2 (7.7)
	Don’t know	8 (34.8)	3 (100)	11 (42.3)
	Missing	10	0	10
	Not applicable	1	1	2
Physical contact with colleagues	No	18 (58.1)	2 (50)	20 (57.1)
	Yes	13 (41.9)	2 (50)	15 (42.9)
	Missing	3	0	3
Divider between colleagues	No	16 (76.2)	1 (33.3)	17 (70.8)
	Yes	5 (23.8)	2 (66.7)	7 (29.2)
	Missing	4	0	4
	Not applicable	9	1	10
Social distance with colleagues	Rarely	6 (20.7)	0	6 (18.2)
	Sometimes	2 (6.9)	0	2 (6.1)
	Mostly	21 (72.4)	4 (100)	25 (75.8)
	Always	0	0	0
	Missing	5	0	5
Lean in	No	7 (21.9)	1 (25)	8 (22.2)
	Yes, sometimes	14 (43.8)	2 (50)	16 (44.4)
	Yes, most of the time	8 (25)	1 (25)	9 (25.0)
	Yes, always	3 (9.4)	0	3 (8.3)
	Missing	2	0	2
Pay change concerns due to work closure	No change	2 (5.9)	2 (50)	4 (10.5)
	Decrease	24 (70.6)	2 (50)	26 (68.4)
	Become zero	1 (2.9)	0	1 (2.6)
	Don’t know	7 (20.6)	0	7 (18.4)
Pay change concerns due to self-isolation	No change	2 (6.1)	3 (75)	5 (13.5)
	Decrease	22 (66.7)	1 (25)	23 (62.2)
	Become zero	1 (3)	0	1 (2.7)
	Don’t know	8 (24.2)	0	8 (21.6)
	Missing	1	0	1
Future income reduction concerns due to self-isolation	No, not at all	5 (14.7)	1 (25)	6 (15.8)
	No, not so much	4 (11.8)	0	4 (10.5)
	Yes, slightly	9 (26.5)	0	9 (23.7)
	Yes, very much	16 (47)	3 (75)	19 (50)
	Not sure	0	0	0
Future unemployment concerns due to self-isolation	No, not at all	11 (32.4)	2 (50)	13 (34.2)
	No, not so much	9 (26.5)	0	9 (23.7)
	Yes, slightly	5 (14.7)	0	5 (13.2)
	Yes, very much	8 (23.5)	0	8 (21.1)
	Not sure	1 (2.9)	2 (50)	3 (7.9)
**COVID-19 prevention**				
COVID-19 training at work	No	1 (3)	0	1 (2.7)
	Yes	32 (97)	4 (100)	36 (97.3)
	Missing	1	0	1
Handwashing or sanitising facilities at work	No	0	0	0
	Yes	32 (100)	4 (100)	36 (100)
	Missing	2	0	2
Good hand hygiene practice signage at work	No	0	0	0
	Yes	31 (100)	4 (100)	35 (100)
	Missing	3	0	3
Vaccinated	No	30 (88.2)	4 (100)	34 (89.5)
	Yes	4 (11.8)	0	4 (10.5)
Vaccine type	Pfizer/bioNTech	1 (25)	0	1 (25)
	Oxford/AstraZeneca	3 (75)	0	3 (75)
	Not applicable	30	4	34
Had a close-contact with symptom/s	No	19 (55.9)	1 (25)	20 (52.6)
	Yes, live with	0	3 (75)	3 (7.9)
	Yes, work with	6 (17.7)	0	6 (15.8)
	Yes, live and work with	0	0	0
	Yes, do not live and work with	0	0	0
	Not sure	9 (26.5)	0	0
Had a close-contact with positive test	No	18 (52.9)	1 (25)	19 (50)
	Yes, live with	0	3 (75)	3 (7.9)
	Yes, work with	9 (26.5)	0	9 (23.7)
	Yes, live and work with	0	0	0
	Yes, do not live and work with	0	0	0
	Not sure	7 (20.6)	0	7 (18.4)

**Table 2:** SARS-CoV-2 RNA results of 36 surface samples taken from various locations following an outbreak at an automotive manufacturing site—England, United Kingdom.

RT-PCR Results (From a Total of 36 Samples)			Level of RNA (Based on Ct Value)		
Confirmed Positive	Suspected Positive	Negative	Moderate–High (Ct < 32.0)	Low (Ct 32.0–34.9)	Very Low–None (Ct ≥ 35.0 ^a^)
14 (38.9%)	3 (8.3%)	19 (52.8%)	0 (0.0%)	2 (5.6%)	34 (94.4%)
**Positive sample information**					
**Site area**	**Location in area**	**Mean Ct value ^b^**		**Estimated copies per cm^2 c^**	
Production lines, assembly, and finishing	Metal storage rack	35.7		5299	
	Work bench	35.6		5195	
	Work bench	37.2 ^d^		1306	
	Work bench	37.9 ^d^		725	
	Cleaning station	36.2		3254	
	Work bench	35.7		4478	
	Work bench	36.1		3385	
	Photocopier	36.4		3863	
Material Preparation	Multi-use bench	37.3		1296	
	Work bench	35.1		7921	
	Metal storage rack	35.3		6537	
	Cupboard	35.9		3835	
Canteen	Vending machine	37.3 ^d^		1186	
Locker room	Locker	35.1		7511	
	Locker	32.7		56,986	
	Locker	36.2		3454	
	Locker	33.1		40,520	

^a^ Includes 19 samples with no SARS-CoV-2 RNA detected. ^b^ Mean Ct value for the N gene. ^c^ Extrapolation from copies per reaction to copies per sample collected based on the dilution factor, then divided by recorded sampling area. ^d^ Sample identified as suspected positive, defined as a sample with a single replicate testing positive for at least one target. Abbreviations: Severe acute respiratory syndrome coronavirus 2 (SARS-CoV-2), Ribonucleic acid (RNA), Real-time polymerase chain reaction (RT-PCR), Crossing threshold (Ct), and Nucleocapsid (N).

## Data Availability

The data that support the findings of this study are available on reasonable request from the corresponding author. The data are not publicly available due to privacy or ethical restrictions.

## Supplementary Materials

The following supporting information can be downloaded at: https://www.mdpi.com/article/10.3390/ijerph19116400/s1, Figure S1: Flow diagram of workers from an automotive manufacturing site participating in COVID-OUT; Table S1: Participant demographic and non-work factor related responses to baseline COVID-OUT study questionnaire.
