# Supplementary material for: Investigation of a SARS-CoV-2 Outbreak at an Automotive Manufacturing Site in England
Source: Int J Environ Res Public Health. 2022 May 24;19(11):6400. doi: 10.3390/ijerph19116400 (PMC9180680; doi:10.3390/ijerph19116400)
Supplement: Supplementary file 1 [file ijerph-19-06400-s001.zip › ijerph-1702189-supplementary.pdf]

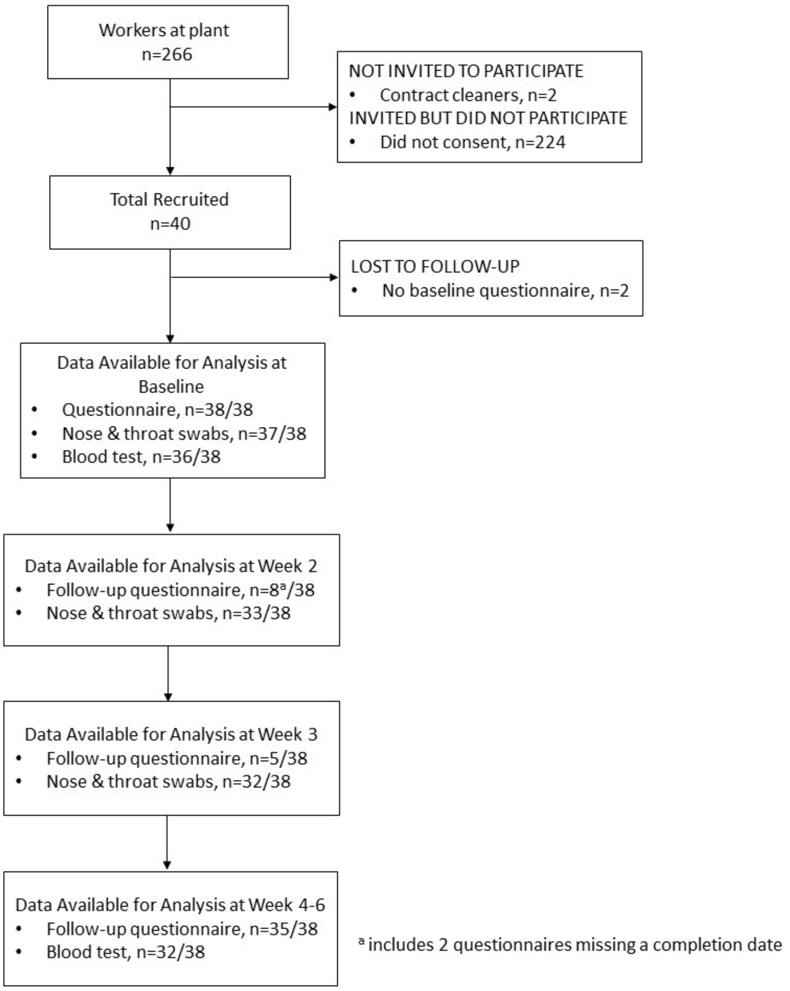

Figure S1. Flow diagram of workers from an automotive manufacturing site participating in COVID-OUT.

Table S1. Participant demographic and non-work factor related responses to baseline COVID-OUT study questionnaire

		Non-cases (n=34)	Cases (n=4)	Total (n=38)
**Demographic information**				
Sex	Male	15 (45.5)	3 (75)	18 (48.6)
	Female	18 (54.5)	1 (25)	19 (51.4)
	Missing	1	0	1
Age	Mean (range)	38.8 (21-54)	52.7 (41-64)	39.7 (21 - 64)
	Missing	0	1	1
Co-morbidities	Diabetes	0	0	0
	High blood pressure	3 (8.8)	1 (25)	4 (10.5)
	Asthma	1 (2.9)	1 (25)	2 (5.3)
	Chronic lung issues	0	0	0
	Chronic heart issues	0	0	0
	Previous stroke	0	0	0
	None reported	30 (88.2)	2 (50)	32 (84.2)
BMI categorical	Healthy weight	12 (36.4)	0	12 (31.6)
	Overweight	9 (27.3)	3 (100)	12 (31.6)
	Obese	12 (36.4)	0	12 (31.6)
	Missing	1	1	2
Smoking status	Never	18 (52.9)	1 (25)	19 (50)
	Former	6 (17.6)	3 (75)	9 (23.7)
	Current	10 (29.4)	0	10 (26.3)
E-cigarettes use	Never	25 (73.5)	3 (75)	28 (73.6)
	Former	4 (11.8)	1 (25)	5 (13.2)
	Current	5 (14.7)	0	5 (13.2)
**Non-work factors**				
Members of household aged ≤19	No	15 (46.9)	3 (100)	18 (51.4)
	Yes	17 (53.1)	0	17 (48.6)
	Missing	2	1	3
Members of household who are co-workers	No	25 (78.1)	1 (33.3)	26 (74.3)
	Yes	7 (21.9)	2 (66.7)	9 (25.7)
	Missing	2	1	3
Visit to shops	No	0	2 (100)	2 (5.9)
	Yes	32 (100)	0	32 (94.1)
	Missing	2	2	4
Visit to place of entertainment	No	32 (100)	3 (100)	35 (100)
	Yes	0	0	0
	Missing	2	1	3
Visit to public buildings	No	26 (83.9)	3 (100)	29 (85.3)
	Yes	5 (16.1)	0	5 (14.7)
	Missing	3	1	4
Visit to educational facilities	No	26 (83.9)	3 (100)	29 (85.3)
	Yes	5 (16.1)	0	5 (14.7)
	Missing	3	1	4
Visit to childcare facilities	No	25 (81.3)	3 (75)	28 (80)
	Yes	7 (21.9)	0	7 (20)

	Missing	2	1	3
Visit to public transport facilities (e.g. stations, airports)	No	32 (100)	3 (100)	35 (100)
	Yes	0	0	0
	Missing	2	1	3
Visit to public transport	No	29 (90.6)	3 (100)	32 (91.4)
	Yes	3 (9.4)	0	3 (8.6)
	Missing	2	1	3
Visit to care home	No	32 (100)	3 (100)	35 (100)
	Yes	0	0	0
	Missing	2	1	3
Visit to private home for socialising	No	29 (90.6)	3 (100)	32 (91.4)
	Yes	3 (9.4)	0	3 (8.6)
	Missing	2	1	3
Visit to private home to care for elderly/ vulnerable adult	No	30 (93.8)	3 (100)	33 (94.3)
	Yes	2 (6.3)	0	2 (5.7)
	Missing	2	1	3
Visit to salon	No	31 (100)	3 (100)	34 (100)
	Yes	0	0	0
	Missing	3	1	4
Visit to indoor fitness/ sport facilities	No	31 (100)	3 (100)	34 (100)
	Yes	0	0	0
	Missing	3	1	4
Visit to park	No	11 (34.4)	3 (100)	14 (40)
	Yes	21 (65.6)	0	21 (60)
	Missing	2	1	3
Visit to doctor	No	20 (62.5)	3 (100)	23 (65.7)
	Yes	12 (37.5)	0	12 (34.3)
	Missing	2	1	3
International travel	No	30 (100)	3 (100)	33 (100)
	Yes	0	0	0
	Missing	4	1	5
Domestic travel	No	28 (100)	3 (100)	31 (100)
	Yes	0	0	0
	Missing	6	1	7
